# Lens and cornea limit UV vision of birds – a phylogenetic perspective

**DOI:** 10.1242/jeb.243129

**Published:** 2021-10-28

**Authors:** Peter Olsson, Olle Lind, Mindaugas Mitkus, Kaspar Delhey, Almut Kelber

**Affiliations:** 1 Department of Biology, Lund University, 22362 Lund, Sweden; 2 Department of Philosophy, Lund University, 22100 Lund, Sweden; 3 Max Planck Institute for Ornithology, 78315 Seewiesen, Germany; 4 School of Biological Sciences, Monash University, 3800 Clayton, Victoria, Australia

**Keywords:** Ultraviolet vision, Spectral sensitivity, Bird visual ecology, Colour vision

## Abstract

Most vertebrates have UV-sensitive vision, but the UV sensitivity of their eyes is limited by the transmittance of the ocular media, and the specific contribution of the different media (cornea, lens) has remained unclear. Here, we describe the transmittance of all ocular media (OMT), as well as that of lenses and corneas of birds. For 66 species belonging to 18 orders, the wavelength at which 50% of light is transmitted through the ocular media to the retina (λ_T0.5_) ranges from 310 to 398 nm. Low λ_T0.5_ corresponds to more UV light transmitted. Corneal λ_T0.5_ varies only between 300 and 345 nm, whereas lens λ_T0.5_ values are more variable (between 315 and 400 nm) and tend to be the limiting factor, determining OMT in the majority of species. OMT λ_T0.5_ is positively correlated with eye size, but λ_T0.5_ of corneas and lenses are not correlated with their thickness when controlled for phylogeny. Corneal and lens transmittances do not differ between birds with UV- and violet-sensitive SWS1 opsin when controlling for eye size and phylogeny. Phylogenetic relatedness is a strong predictor of OMT, and ancestral state reconstructions suggest that from ancestral intermediate OMT, highly UV-transparent ocular media (low λ_T0.5_) evolved at least five times in our sample of birds. Some birds have evolved in the opposite direction towards a more UV-opaque lens, possibly owing to pigmentation, likely to mitigate UV damage or reduce chromatic aberration.

## Abstract (precis)

**Summary:** UV sensitivity in bird eyes is determined by the transmittances of the cornea and lens. Pigmentation could explain some of the variation. Phylogenetically related birds are likely to have similar transmittance.

## INTRODUCTION

The majority of animals, including most vertebrates, can see ultraviolet (UV) light, with wavelengths shorter than 400 nm (for a review, see Cronin and Bok, 2016). Two conditions must be fulfilled for a vertebrate visual system to be UV-sensitive: it needs to possess UV-sensitive photoreceptors and UV-transmitting ocular media, including cornea, aqueous humour, lens and vitreous humour.

In most birds, colour vision is based on four types of cone photoreceptors, expressing visual pigments sensitive to long (LWS, peak sensitivity 560–570 nm), medium (MWS, 497–509 nm), short (SWS2, 427–458 nm) and very short (SWS1, 355–426) wavelengths (Hart, 2001; Hart and Hunt, 2007). The spectral sensitivities of the LWS and MWS visual pigments vary little among birds, but SWS1 and SWS2 are more variable (Kelber, 2019). Although owls and some other birds have lost SWS1 (Höglund et al., 2019; Kelber, 2019), most birds can be categorized as either ultraviolet-sensitive (UVS), with a UV-sensitive SWS1-based visual pigment maximally sensitive to wavelengths between 355 and 373 nm, or violet-sensitive (VS), with SWS1 sensitivity peaking in the violet range between 399 and 425 nm (Hart, 2001; Ödeen and Håstad, 2003; Håstad et al., 2005a; Ödeen and Håstad, 2013).

For birds to fully utilise the retinal UV sensitivity, their ocular media must transmit light of wavelengths below 400 nm. All ocular media of birds have high transmittance for long-wavelength light (400–700 nm), and the humours transmit light of wavelengths down to 300 nm (Douglas and Marshall, 1999; Zawadzka et al., 2021). Light of wavelengths close to 300 nm is absorbed by nucleic acids and amino acids (e.g. Douglas and Marshall, 1999) and scattered by structural elements such as collagen fibrils in the cornea (Tsukahara et al., 2010; Meek and Knupp, 2015). Because light is inevitably scattered and absorbed on its path through the eye, ocular media transmittance (OMT) depends on the axial length of the eye in birds (Lind et al., 2014; Olsson et al., 2016) as well as in mammals (Douglas and Jeffery, 2014) and some fishes (Thorpe and Douglas, 1993). Thus, UVS birds are generally smaller and have more transparent ocular media than VS birds (Lind et al., 2014).

In some bird species, including raptors and swifts, the ocular media transmit less UV than expected from eye size (Lind et al., 2014). This observation could possibly be explained by these birds having (i) relatively thick lenses and corneas, (ii) higher structural disorder in the ocular media and thus increased scatter (see, for instance, Tsukahara et al., 2010) or (iii) UV-absorbing pigments in the lens and/or cornea. Pigmented lenses have been found in fishes, lizards and some mammals including humans, either by direct identification (Douglas and Marshall, 1999; Röll, 2000; Douglas and Jeffery, 2014) or by inference from low lenticular UV transmittance (Siebeck and Marshall, 2001, 2007). Corneal pigmentation is common in fishes (Douglas and Marshall, 1999; Siebeck and Marshall, 2001, 2007) but is assumed to be absent in terrestrial vertebrates (Douglas and Marshall, 1999).

In this study, we present total OMT (*n*=66 species, including 30 newly measured species) as well as the transmittances of corneas (*n*=41 species) and lenses (*n*=51 species) of birds. We asked: (i) which ocular medium – lens or cornea – limits OMT of birds, and (ii) to what degree does the transmittance of the ocular media depend on the type of visual system (VS or UVS), eye size and phylogeny. We also aimed to reconstruct the evolution of whole ocular media, lens and cornea transmittance in birds.

## MATERIALS AND METHODS

### Animals

Most eyes were collected from severely injured birds that had to be euthanized in an animal rescue centre in southern Sweden. The eyes of European honey buzzard (*Pernis apivorus*), red kite (*Milvus milvus*), common kestrel (*Falco tinnunculus*), western marsh harrier (*Circus aeruginosus*) and white-tailed eagle (*Haliaeetus albicilla*) were collected with permission from national Swedish authorities (Naturvårdsverket, NV-03136-14). Common ostrich (*Struthio camelus*), Japanese quail (*Coturnix japonica*) and domestic chicken (*Gallus gallus domesticus*) eyes were collected from animals euthanized for reasons unrelated to this study; the collection of common ostrich eyes was approved by local authorities (Jordbruksverket 6.2.18-8245/13). For some analyses, we combine newly collected data with previously published data (see Table 1 for references).

**Table 1.**
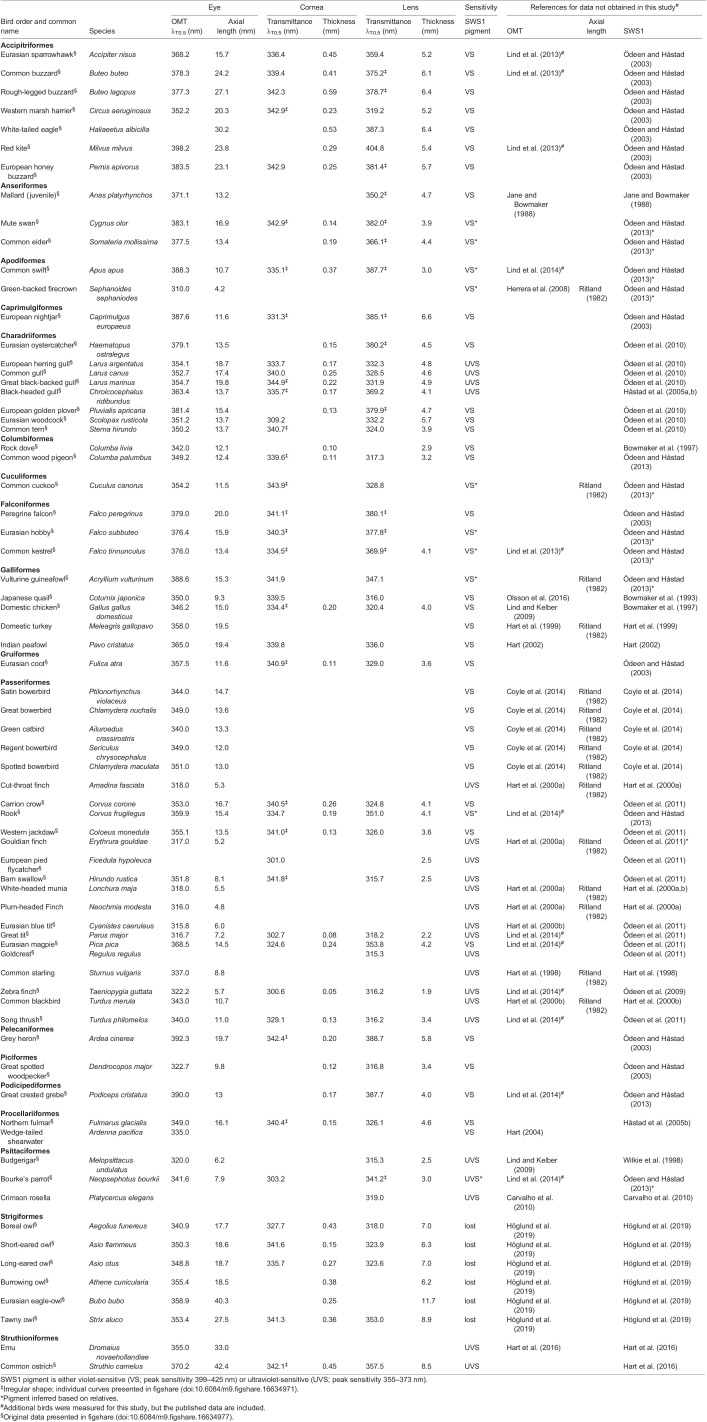
Eye length, lens and corneal thickness as well as the λ_t50_, and the state of SWS1 opsin (UVS or VS) of all bird species included in this study. Bird orders and species are listed in alphabetical order.

### Measurement of transmittance

OMT was measured as described previously (Lind et al., 2013; Olsson et al., 2016). The eye was enucleated and on the posterior pole of the eye, the sclera, choroid, retinal pigment epithelium and retina were removed carefully. The eye was then placed, with the cornea pointing downwards, in a custom-made matte black cylindrical container with a silica window in the bottom, filled with a 340 mOsm kg^−1^ phosphate buffered saline (PBS), pH 7.2. Light from a PX2 Xenon lamp (Ocean Optics, Dunedin, FL, USA) illuminated the eye from the corneal side, through a 1 mm wide light guide (UV-VIS, Ocean Optics). Transmitted light was collected by an identical light guide and measured with 1 nm resolution, using a Maya 2000 spectroradiometer (Ocean Optics) controlled by Spectrasuit software (v 1.0, Ocean Optics). After measuring total OMT, the cornea and lens were extracted and measured in the same setup.

**Fig. 1.**
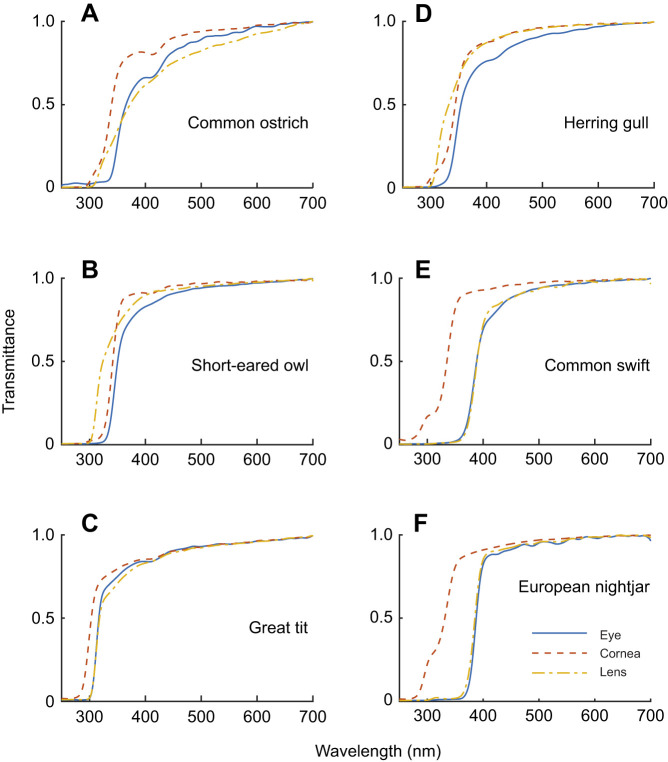
**Ocular media transmittance (OMT) of birds.** (A–E) Average transmittance functions of the ocular media (blue solid lines), cornea (red dashed lines) and lens (yellow dot-dashed lines) of (A) common ostrich, (B) short-eared owl, (C) great tit, (D) herring gull, (E) common swift and (F) European nightjar. All measured curves are presented in the deposited data (data, doi:10.6084/m9.figshare.16634977; figures, doi:10.6084/m9.figshare.16634971).

All spectral transmittance functions were normalized to the maximum value between 250 and 700 nm and smoothed by 11 nm moving averages (MATLAB 2012–2015a). For each eye, cornea or lens, the average transmittance function is based on three to eight measurements. For each species, we determined the average transmittance curve of all available specimens. From these average curves, we determined the wavelength at which 50% of the light was transmitted (λ_T0.5_; Lind et al., 2014). To allow for comparison with other data sets, we also calculated the percentage of UVA (315–400 nm) reaching the retina (see Douglas and Jeffery, 2014). For higher λ_T0.5_, less UV radiation is transmitted to the retina. We present all transmittance functions in the deposited data (data, doi:10.6084/m9.figshare.16634977; figures, doi:10.6084/m9.figshare.16634971). Transmittance functions are most variable in the UV range; to illustrate this fact better, we plotted the cornea and lens functions between 250 and 400 nm, normalised to the transmittance value at 400 nm. Note that all reported λ_T0.5_ are from transmittance functions normalised to maximum transmittance between 250 and 700 nm. When the transmittance function of a single specimen within one species showed an irregularity in the transmittance function (e.g. Fig. 1E), we noted this for the species in general in Table 1 (marked with ‡).

### Size measurements

Eye size was measured as described previously (Lind et al., 2014; Mitkus et al., 2018). The eye or head of a freshly dead bird was frozen at −80°C and sectioned horizontally in a cryotome (HM-560, Thermo Fisher Scientific, Waltham, MA, USA). Photographs (Canon EOS 500D camera with a Canon Ultrasonic 100 mm macro objective) of the head or eye block were taken every 100 or 150 μm, with a ruler at the same distance from the camera serving as a scale. We measured eye dimensions from the photograph featuring the largest pupil and longest lens path length, using ImageJ (Schneider, 2012). Eye size was measured as the axial length from the cornea apex to the back of the sclera. Corneal and lens thickness were measured along this line. For additional species, we used axial length measurements from Ritland (1982).

### Statistical analyses

All analyses were carried out in the R statistical environment (https://www.r-project.org/). We used phylogenetic linear models to assess the effects of predictors (eye size, thickness of cornea and lens, UVS/VS visual system) on the transmittance of the total ocular media, cornea and lens as implemented by function ‘pgls’ in the package ‘caper’ (https://CRAN.R-project.org/package=caper). All models were repeated across a sample of 1000 phylogenies downloaded from the birdtree.org website (Jetz et al., 2012) to account for phylogenetic uncertainty. The results were combined using a model averaging approach (Symonds and Moussalli, 2011). We used published data on VS or UVS visual systems (Aidala et al., 2012; Håstad et al., 2005a,b; Ödeen et al., 2010, 2011; Ödeen and Håstad, 2013); in 10 species no information was available, and we inferred the visual system from closely related species (Table 1, marked with *).

To reconstruct ancestral traits at the root of the phylogeny for the transmittance of the total ocular media, cornea and lens and assess the direction of changes during evolution, we used the function ‘fastAnc’. We estimated phylogenetic signal (Pagel's lambda) using the function ‘phylosig’ from the package ‘phytools’ (Revell, 2012). Again, to account for phylogenetic uncertainty, we repeated these analyses across the 1000 phylogenies and report the mean values.

## RESULTS

### Total OMT

**Fig. 2.**
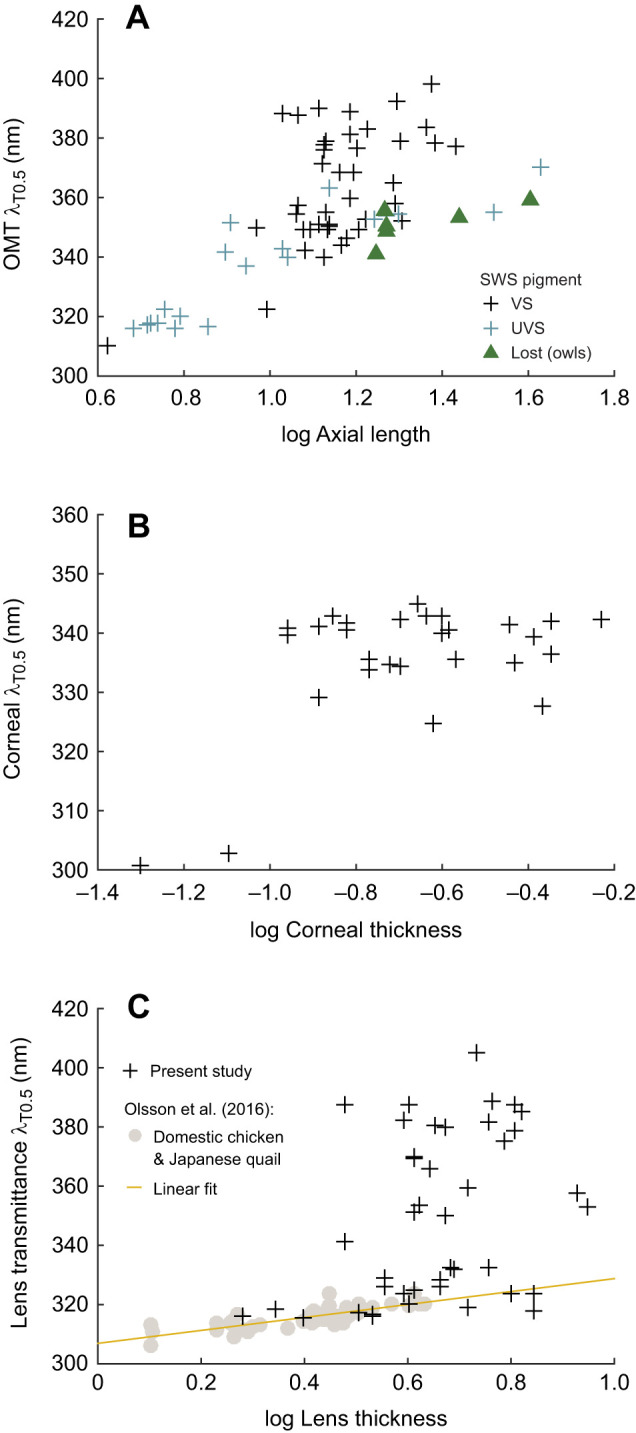
**OMT of birds as a function of size.** (A) The average λ_T0.5_ of total OMT as a function of the axial length (mm) of the eye. *n*=67 species. UVS: species with peak sensitivity of SWS1 opsin between 355 and 370 nm. VS: species with peak sensitivity of SWS1 opsin between 400 and 425 nm. Lost: absence. (B) Corneal λ_T0.5_ as a function of corneal thickness (mm). *n*=27 species. (C) Lens λ_T0.5_ as a function of lens thickness (mm). *n*=43 species. The data from domestic chicken and Japanese quail lenses (grey filled circles; Olsson et al., 2016) and the relationship between lens thickness and lens λ_T0.5_ established from these data (solid line) are given for reference. Note that the abscissae use a logarithmic scale.

**Fig. 3.**
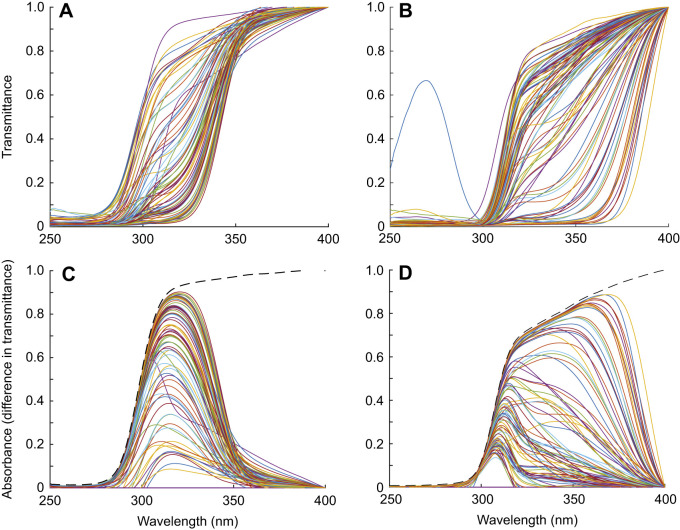
**Transmittance functions of individual corneas and lenses.** The transmittance functions of all corneas (A) and lenses (B) of all individual birds measured in the UV range. The transmittance below 300 nm for one lens is due to an artifact in that specific range only. Difference spectra for all individual corneas (C) and lenses (D) compared with the most transmissive cornea and lens, respectively (marked with dashed lines).

**Fig. 4.**
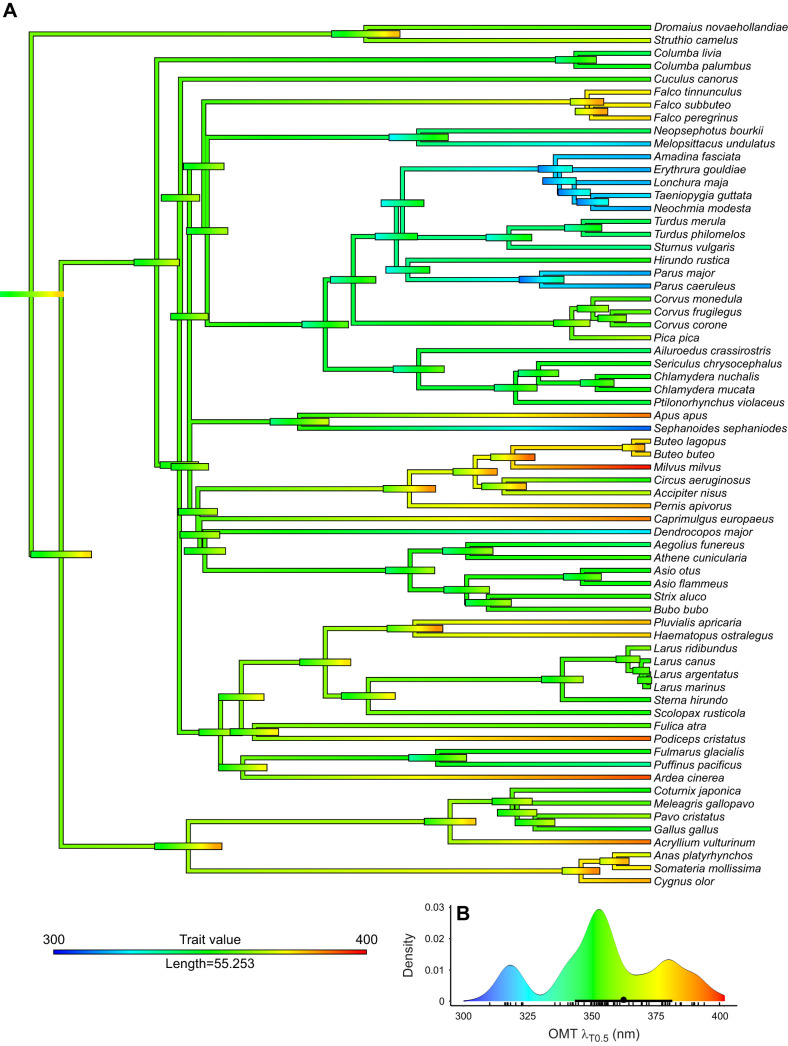
**Ancestral reconstruction and evolution of OMT.** (A) The bars at the nodes correspond to uncertainty of the estimated value at that node. The width of the bars corresponds to the degree of uncertainty and the colour reflects the trait value according to the inset. (B) Distribution of OMT and ancestral OMT [average (circle) with dark bar representing 95% confidence interval] at the base of the phylogeny (root). Each small vertical line at the *x*-axis represents one species average.

We include data from 71 species belonging to 18 orders in the analyses (Table 1, and https://figshare.com/articles/dataset/Supplementary_data/16634971). Of these, we present new measurements from the OMT of 30 species of birds (Fig. 1A–E) and used published data on 40 additional species. In some cases, we added measurements of new individuals to species previously measured. For four species, OMT could not be determined. The ocular media of all included birds were highly transmissive between 400 and 700 nm, but varied considerably in the UV part of the spectrum, with λ_T0.5_ ranging from 310 to 398 nm (Table 1, Figs 2A, 3A,B). We can roughly categorise birds into three groups, with low, intermediate and high λ_T0.5_ (Fig. 4B).

We included some estimates of variation between individuals of the same species (Table 2). Note that these averages are of the extracted λ_T0.5_ values from individuals, not from an average curve as in Table 1. For the great tit, Bourke's parrot and rook, there were noticeable variance. In the case of the great tit and rook, it seems to stem from some individuals that showed higher scatter. In the case of the Bourke's parrot, one individual had much lower OMT for unknown reasons

**Table 2.**
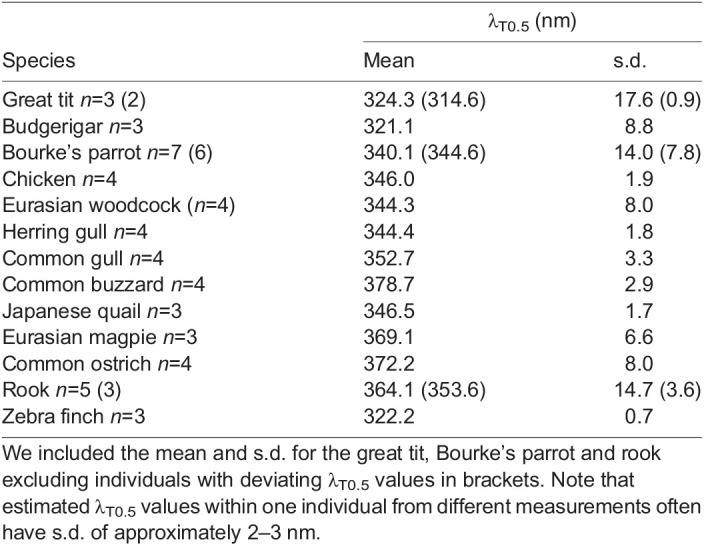
Mean λ_T0.5_ values and standard deviations

The percentage of UVA (315–400 nm) reaching the retina is given in the deposited data (doi:10.6084/m9.figshare.16634977) to allow for comparison with other data sets. This value clearly shows that even in eyes with a high λ_T0.5_ of OMT, some UV radiation reaches the retina.

Ancestral trait reconstruction (Fig. 4A) suggests that eyes with high and low λ_T0.5_ evolved repeatedly from an ancestor with an estimated λ_T0.5_ at 361.4 nm (95% CI: 342.6–380.1). Notably, low λ_T0.5_ values are evident in passerines, parrots, a hummingbird and a woodpecker (Fig. 4). Closely related species had similar λ_T0.5_ values (trait phylogenetic signal Pagel's lambda: 1). Birds with smaller eyes (log_10_ axial length of the eye) had lower λ_T0.5_ values (effect: 45.624, s.e.: 12.351, *P*<0.0001; Table S2) and so did UVS birds. However, the latter effect is not statistically significant when we account for the effect of eye size (VS–UVS, effect: 9.597, s.e.: 5.791, *P*=0.102; Table S2). This result does not depend on whether owls (diamonds in Fig. 2A) are included as deriving from a lineage of VS species, or as having lost the SWS1 pigment (Tables S2 and S3).

### Transmittance of lenses and corneas

**Fig. 5.**
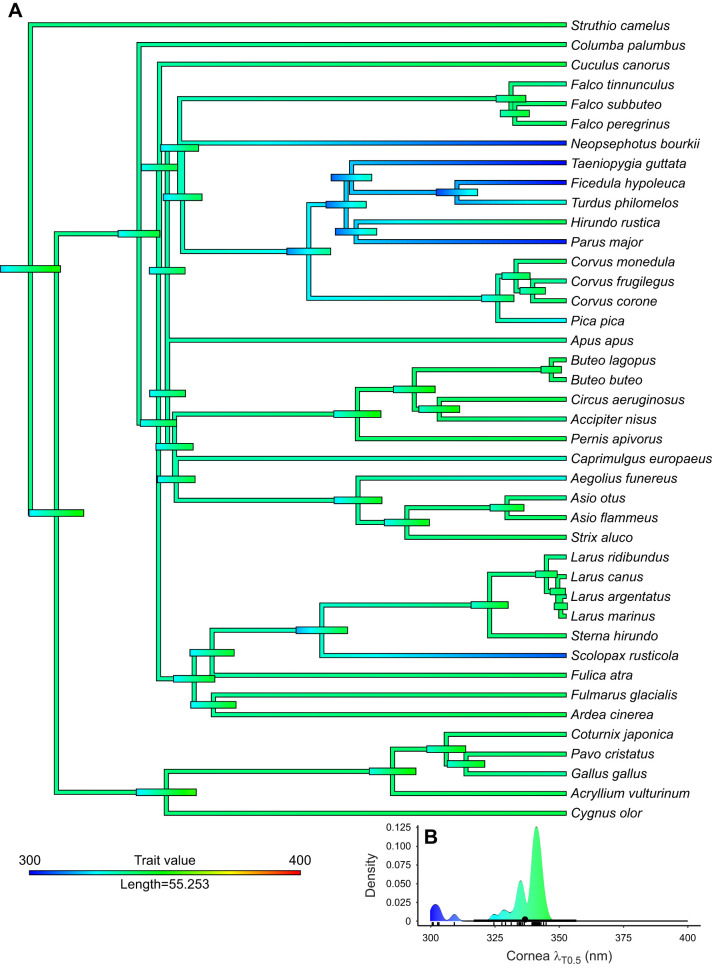
**Ancestral reconstruction and evolution of corneal transmittance.** (A) The bars at the nodes correspond to uncertainty of the estimated value at that node. The width of the bars corresponds to the degree of uncertainty and the colour reflects the trait value according to the inset. (B) Distribution of corneal transmittance and ancestral corneal transmittance [average (circle) with dark bar representing 95% confidence interval] at the base of the phylogeny (root). Each small vertical line at the *x*-axis represents one species average.

**Fig. 6.**
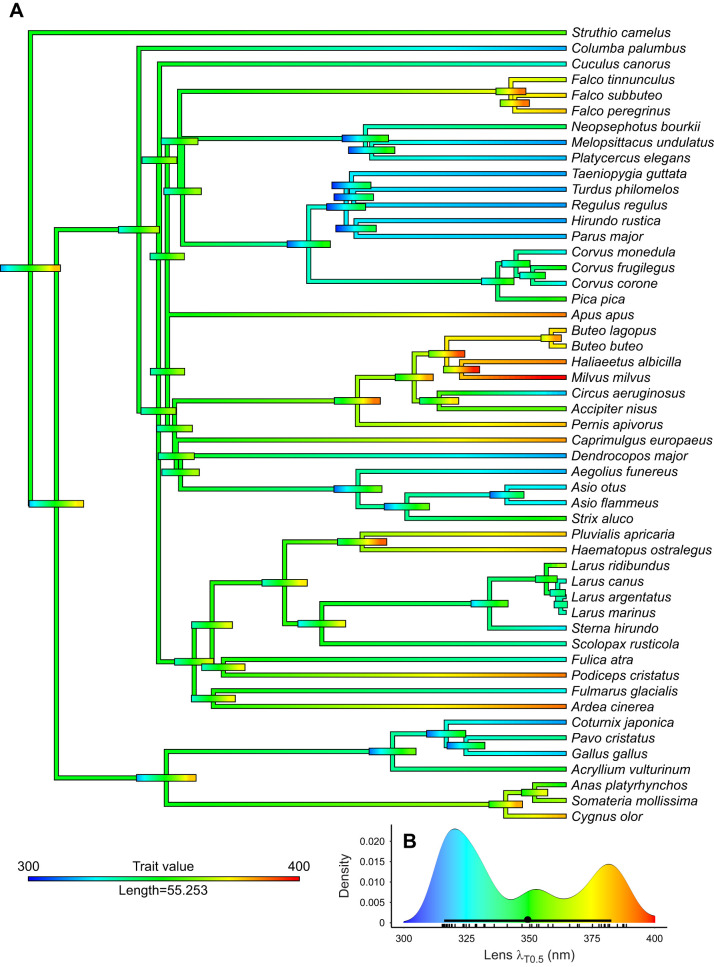
**Ancestral reconstruction and evolution of lens transmittance.** (A) The bars at the nodes correspond to uncertainty of the estimated value at that node. The width of the bars corresponds to the degree of uncertainty and the colour reflects the trait value according to the inset. (B) Distribution of lens transmittance and ancestral lens transmittance [average (circle) with dark bar representing 95% confidence interval] at the base of the phylogeny (root). Each small vertical line at the *x*-axis represents one species average.

The transmittance functions of lenses (51 species) and corneas (41 species) are available in the deposited data (doi:10.6084/m9.figshare.16634977) whereas individual transmittance functions in the UV range are available in Fig. 3. Corneas of all studied birds were highly transmissive down to 350 nm, and λ_T0.5_ of corneal transmittance varied only between 300 and 345 nm (Figs 1–3, Table 1), whereas lens λ_T0.5_ varied more widely between 315 and 400 nm (Figs 1–3, Table 1). Ancestral trait reconstructions gave a mean λ_T0.5_ of 336.15 nm as the likely ancestral trait for the cornea (95% CI: 315.20–357.11; Fig. 5) and a mean λ_T0.5_ of 349.38 nm for the lens, but the estimate had a very high uncertainty (95% CI: 316.06–382.69; Fig. 6). In both cases, closely related species often had similar λ_T0.5_, indicating a strong phylogenetic signal (phylogenetic signal Pagel's lambda, cornea: 0.89; lens: 0.86).

Corneal thickness was measured in 27 species. The corneas of all studied birds were thin, measuring between 0.05 mm (zebra finch, *Taeniopygia guttata*) and 0.6 mm (rough-legged buzzard, *Buteo lagopus*). Even though the two thinnest corneas in our sample, those of the zebra finch and great tit (*Parus major*), had the highest corneal transmittance, there was no significant relationship between corneal λ_T0.5_ and corneal thickness (effect: 13.211, s.e.: 16.345, *P*=0.427; Fig. 2B, Table S4). Corneal transmittance did not differ significantly between UVS and VS species (VS-UVS, effect: 0.452, s.e.: 5.51, *P*=0.896, Table S4). In the corneal transmittance functions of several species, an irregular ‘bump’ was observed between 300 and 320 nm (Figs 1, 3).

For our sample of lenses (lens thickness was determined in 43 species), λ_T0.5_ did not correlate with lens thickness (effect: 4.487, s.e.: 3.116, *P*=0.157; Fig. 2C, Table S5), and did not differ significantly between UVS and VS species (effect: 15.380, s.e.: 10.180, *P*=0.139). We can roughly identify three groups of species with low, intermediate and high λ_T0.5_ (Fig. 6B). The variation in lens λ_T0.5_ was associated with differences in the shape of the transmittance functions. Several lenses with intermediate λ_T0.5_ had more irregular shapes with a wide trough centred at approximately 340 nm (Figs 1G, 3B). The transmittance functions of lenses with high λ_T0.5_ (>360 nm) remained quite low between 300 and 350 nm and then increased steeply until approximately 390–400 nm (Fig. 3B).

### Contribution of cornea and lens to OMT

**Fig. 7.**
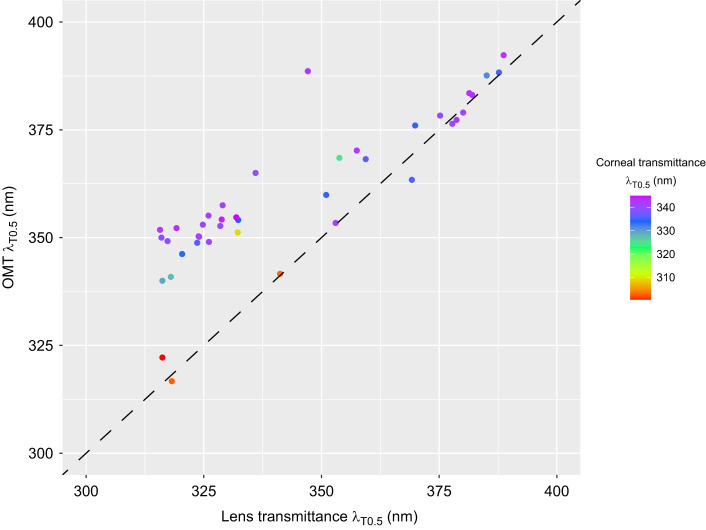
**Relationship between total OMT and the lens and corneal transmittances.** Corneal transmittance values are represented in colour. The included line refers to the expectation that OMT and lens transmittance are equal.

In most species, including the common ostrich (Fig. 1A), the great tit (Fig. 1C) and the common swift (*Apus apus*) (Fig. 1E), the cornea (dashed line) transmits light of shorter wavelengths than the lens (dotted lines), thus the lens has the strongest impact on OMT. Only in a few species, including the short-eared owl (*Asio flammeus*) (Fig. 1B) and the Japanese quail, does the cornea limit OMT. If we assess their contributions separately, the lens generally has a stronger influence on OMT (effect: 0.585, s.e.: 0.069, *P*<0.0001; Table S7) than the cornea (effect: 0.227, s.e.: 0.156, *P*=0.148; Table S6). Together, lens transmittance (effect: 0.538, s.e.: 0.045, *P*<0.0001) and cornea transmittance (effect: 0.569, s.e.: 0.113, *P*<0.0001) explain more than 80% of the variation in total OMT when included in the same model (Table S8). Accordingly, there is a linear relationship between λ_T0.5_ of total OMT and λ_T0.5_ of the lens (Fig. 6). Several species have higher λ_T0.5_ of OMT than expected from that relationship, and these species all have intermediate λ_T0.5_ of OMT and relatively high λ_T0.5_ of the cornea (330–340 nm; Fig. 7).

## DISCUSSION

### High variation in OMT

We found high variation in the total OMT and lens transmittance amongst our sample of birds. OMT is correlated with eye size, but as observed earlier (Lind et al., 2014), many birds have lower OMT (higher λ_T0.5_) than expected from eye size. All birds with highly UV-transmissive ocular media (λ_T0.5_ <325 nm), which include small passerines, the great spotted woodpecker (*Dendrocopus major*), the budgerigar (*Melopsittacus undulatus*) and a hummingbird (green-backed firecrown, *Sephanoides sephaniodes*), have very small eyes (axial length <10 mm). However, some bird species with moderately sized eyes (axial length 10–20 mm), including several raptors (order Accipitriformes), the common swift, the European nightjar (*Caprimulgus europaeus*), the mute swan (*Cygnus olor*), the golden plover (*Pluvialis apricaria*), the great crested grebe (*Podiceps cristatus*) and the grey heron (*Ardea cinerea*), have λ_T0.5_ of 380 nm or higher, while the birds with the largest eyes in our sample, the Eurasian eagle owl (*Bubo bubo*) and the common ostrich (axial length >40 mm), have comparatively low λ_T0.5_ of 359 and 370 nm, respectively (Table 1).

With the exception of a small number of species with very small eyes, corneal transmittance is quite similar among the studied birds. Regardless of corneal thickness or eye size, corneal λ_T0.5_ is lower than total OMT λ_T0.5_. None of the birds had a corneal λ_T0.5_ higher than 345 nm (Fig. 2B). Thus, corneal transmittance can be excluded as a reason for the unexpectedly low OMT and high λ_T0.5_ of total OMT in some birds.

### Lens transmittance limits UV vision in most birds

In most bird eyes, the lens limits OMT, specifically in species with λ_T0.5_ higher than expected from eye size. We included data from chickens and quails of different age and size (Olsson et al., 2016; grey symbols and regression line in Fig. 2C) as a proxy for the relationship between the thickness and the transmittance (λ_T0.5_) of unpigmented lenses. A similar relationship has been found in a sample of unpigmented fish lenses (Thorpe and Douglas, 1993). If all bird lenses included in our study were unpigmented, we would expect a similar relationship of transmittance and lens thickness as that found for the unpigmented lenses of chickens, quails and fish. However, many lenses transmit far less UV light than expected from their size. Most diurnal raptors (orders Falconiformes and Accipitriformes, excluding the western marsh harrier), many anseriforms, some charadriiforms, the grey heron, the Eurasian swift, the European nightjar, the Eurasian magpie (*Pica pica*) and the great crested grebe fall into this group.

In many fishes (Douglas and McGuigan, 1989; Thorpe et al., 1993; Douglas and Marshall, 1999; Siebeck and Marshall, 2001) and some frogs (Yovanovich et al., 2020), irregularities or ‘bumps’ in the transmittance curves indicate which lens pigments absorb light in the UV range. We find similar bumps in the transmittance curves of approximately 17 bird lenses (Fig. 1G, Table 1, marked with ‡; also see doi:10.6084/m9.figshare.16634977, doi:10.6084/m9.figshare.16634971), and suggest that such bird lenses with low transmittance (thus, high lens λ_T0.5_) may also be pigmented (Lind et al., 2014). However, to confirm this, chemical analyses are required. We calculated the difference spectrum between the most transmissive lens in the data set and all other lenses to find the wavelength range where the largest difference can be found. This showed a relatively broad band of reduced transmittance with a peak close to 350 nm (Fig. 3D).

Corneal transmittance is generally not well understood (Piatigorsky, 1998). The thickest layer of the cornea, the stroma, mainly consists of keratocytes and collagen fibrils. In humans, the stroma has a dominant role for corneal transmittance (Kolozsvári et al., 2002). Variation of corneal transmittance among terrestrial vertebrates has been linked to differences in the density and diameters of collagen fibrils, the predominant scatterers in the cornea, as well as the spatial order of the fibrillary arrays (Tsukahara et al., 2010, 2014). However, the thin epithelial layers of the bird cornea also contain high concentrations of proteins including tau-crystallin and cyclophilin that might influence transmittance (Piatigorsky, 1998). Interestingly, λ_T0.5_ of bird corneas tends to be higher than in mammals (Tsukahara et al., 2014). We found bumps in the transmittance curves of 20 bird corneas (Figs 1E, 3A, Table 1 marked with ‡‡; doi:10.6084/m9.figshare.16634971). We calculated the difference spectrum between the most transmissive cornea in the data set and all other corneas to find the wavelength range where the largest difference can be found. This showed a narrow band of lower transmittance with a peak at 315–320 nm (Fig. 3C), consistent with what we found with chicken and quail previously. We have tried to chemically identify pigments in chicken corneas, but this search did not lead to any result (P.O. and A.L., unpublished data). However, we cannot exclude a contribution of pigments to UV absorption in bird corneas.

Studies of the structural and chemical properties of the lenses and corneas of birds, including their proteins, would be highly relevant to test whether absorbing pigments or scatter could explain the shapes of the transmittance curves. Corneal structure has only been documented in very few species, and the crystallins of birds are severely understudied and probably more variable than known to date (Piatigorsky, 1998; Tsukahara et al., 2010, 2014).

### OMT and the ecological context of UV vision

Among the studied birds, we would like to point out some interesting observations. The finding that the correlation between the type of the SWS1 opsin – UVS versus VS – and OMT is not significant when phylogeny and eye size are taken into account seems to contradict earlier results (Lind et al., 2014). It likely indicates that UV sensitivity of the SWS1 pigment has evolved more often in bird lineages with small body size, and thus, the small eye size explains their high OMT.

In this context, it may be important to point out that all transmittance curves have sigmoid shapes, thus bird retinas still receive a relatively high intensity of light with wavelengths below the λ_T0.5_, at least in bright light when they use cone-based colour vision. For instance, despite its seemingly high λ_T0.5_ of 370 nm, over 30% of UVA (light of wavelengths between 315 and 400 nm) reaches the retina of the common ostrich, which has UV-sensitive SWS1 opsin (see doi:10.6084/m9.figshare.16634977). Höglund et al. (2019) concluded that the high UV transmittance of owl ocular media is probably related to the need of these predominantly nocturnal birds to catch as many photons as possible for rod vision, as Strigiformes has lost the SWS1 opsin and with it the UV/V sensitivity in daylight. The exact modelling of light received from a visual scene, with methods such as those proposed by Tedore and Nilsson (2019), is required for a better understanding of the ecological relevance of OMT in each species.

### Phylogeny and comparison with other vertebrates

The evolutionary background of a given bird species appears to be a strong predictor of its OMT; related species tend to have ocular media with similar transmittance. OMT is used to estimate the spectral sensitivity of bird vision, and the strong phylogenetic signal that we find in our data indicates that it is often legitimate to use OMT data from closely related species, if this information is not available for the species under study. However, some examples, such as the low λ_T0.5_ of the western marsh harrier and the large variation among charadriiforms, show that such generalization may still lead to mistakes in some cases.

The strong phylogenetic signal of bird OMT is reminiscent of other vertebrates. Among mammals, nocturnal rodents share high lens transmittances, at least while they are young (Douglas and Jeffery, 2014), and the transmittance of frog lenses also indicate a high phylogenetic signal (Yovanovich et al., 2020). This pattern may, however, be just an effect of the relatively small number of studied species: we have studied 67 out of more than 10,000 species of birds, and do not cover all bird orders. Douglas and Jeffery (2014) present lens transmittances of 38 species of mammals, and Yovanovich et al. (2020) have studied 37 species of frogs. OMT is also known for 18 species of snakes (Simões et al., 2016), whereas fishes are the best-studied vertebrates in this respect (>200 species; Thorpe et al., 1993; Douglas and Marshall, 1999; Siebeck and Marshall, 2001, 2007). More studies on additional species are required for a general understanding of ecological, phylogenetic and other constraints on OMT in birds and other vertebrates.

Birds are similar to other tetrapods such as frogs (Yovanovich et al., 2020) and mammals (Kolozsvári et al., 2002; Douglas and Jeffery, 2014; Tsukahara et al., 2014) in having highly UV-transparent corneas, unlike many species of fish (Douglas and Marshall, 1999). Regarding the lens transmittance – which limits total OMT in most cases – birds differ from other vertebrate classes. Even though some bird species have OMT λ_T0.5_ values close to 400 nm, we have not found any species with a lens absorbing light of wavelengths longer than 400 nm. By contrast, more than half of over 200 species of fish (Siebeck and Marshall, 2001, 2007), half of the 38 investigated species of mammals (Douglas and Jeffery, 2014), one-third of the studied snakes (Simões et al., 2016) as well as several species of diurnally active frogs (Yovanovich et al., 2020) have lens λ_T0.5_ values greater than 400 nm. High UV transmittance and UV sensitivity is generally common in nocturnal vertebrates, whereas diurnal species more often have pigmented lenses absorbing well into the violet and blue range of the spectrum (see Yovanovich et al., 2020). No birds – most of which are diurnal – seem to cut out this part of the spectrum. On the one hand, this part of the spectrum seems to be too important for birds to sacrifice it, but on the other hand, they must also have means to protect the lens – perhaps by less cataract-prone types of crystallines – and retina from UV damage (see Carvalho et al., 2010 for discussion). However, cataracts are known from many species of birds (e.g. Keymer, 1977; Galván et al., 2012).

The ancestral reconstructions allow us to speculate how OMT may have evolved. Given that the common ostrich – the phylogenetically most basal bird in our sample – has large eyes and an intermediate λ_T0.5_, an unpigmented lens is the most likely ancestral state. The ancestral state of the SWS1 opsin seems to be UV sensitivity (Hart et al., 2016), and violet and UV sensitivity have evolved repeatedly (Ödeen and Håstad, 2013). From this hypothetical ancestral state – intermediate OMT with an unpigmented lens and a UVS visual pigment – two directions have been taken by different groups of birds. First, low OMT (high λ_T0.5_) evolved repeatedly, probably by the deposition of UV-absorbing pigments in the lens, independent of eye size, and with two main effects: protection from UV damage, and reduction of chromatic aberration (Douglas and Marshall, 1999; Douglas and Jeffery, 2014). This configuration is often combined with VS visual pigment. Second, small birds with small eyes, and thus highly UV-transmissive lenses may have facilitated the evolution of both UVS pigments and structural or chemical changes that make the cornea more UV-transmissive. Ocular media with high UV transmittance allow the detection of UV radiation (e.g. Tedore and Nilsson, 2019) and may provide a ‘private’ communication channel among birds such as blue tits (*Cyanistes caeruleus*) that will not facilitate detection from UV-blind diurnal raptors (Håstad et al., 2005a,b; Lind et al., 2013, 2014).

In summary, to what degree the sensitivity of the SWS1 visual pigment co-evolved with OMT needs to be elucidated by investigations of both features in more species of birds. For a clearer understanding of the evolution and the ecological relevance of OMT in birds, the identification of potential lens pigments, lens proteins (crystallins) and corneal structure is also highly desirable.

## Supplementary Material

Supplementary information
