# Supplementary material for: Lens and cornea limit UV vision of birds – a phylogenetic perspective
Source: J Exp Biol. 2021 Oct 28;224(20):jeb243129. doi: 10.1242/jeb.243129 (PMC8601714; doi:10.1242/jeb.243129)
Supplement: Supplementary information [file jexbio-224-243129-s1.pdf]

Table S1. The dependence of ocular media $\lambda_{T0.5}$ on axial length of the eye (N = 66 species).

variable	estimate	SE	p.value	lambda	r2	r2.adj
**intercept**	304.610	17.674	<0.0001	1	0.183	0.170
**log10(axial.length)**	46.711	12.535	**0.0004**			

Table S2. The dependence of ocular media $\lambda_{T0.5}$ on axial length of the eye and the type of visual system (VS or UVS SWS1 opsin), including owls with the VS birds, from which they likely descended (N = 66 species).

variable	estimate	SE	p.value	lambda	r2	r2.adj
**intercept**	299.242	17.703	<0.0001	1	0.217	0.193
**log10(axial.length)**	45.624	12.351	**<0.0001**			
**visual.system v**	9.597	5.791	0.102			

Table S3. The dependence of ocular media $\lambda_{T0.5}$ on axial length of the eye and the type of visual system (VS or UVS SWS1 opsin), excluding owls, which lack SWS1 opsin (N = 60 species).

variable	estimate	SE	p.value	lambda	r2	r2.adj
**intercept**	293.198	19.175	<0.0001	1	0.238	0.211
**log10(axial.length)**	51.048	13.704	**<0.0001**			
**visual.system v**	9.989	5.957	0.099			

Journal of Experimental Biology • Supplementary information

Table S4. The relationship between cornea thickness and cornea transmittance ($\lambda_{T0.5}$) and type of visual system (VS or UVS).

**variable**	**estimate**	***SE***	**p.value**	**lambda**	**r2**	**r2.adj**
**intercept**	333.64	7.57	<0.0001	0.832	0.032	-0.046
cornea.thick	13.211	16.345	0.427	0.832		
visual.systemv	0.452	5.51	0.896	0.832		

Table S5. The relationship between lens thickness and lens transmittance ($\lambda_{T0.5}$) and type of visual system (VS or UVS).

**variable**	**estimate**	**SE**	**p.value**	**lambda**	**r2**	**r2.adj**
**intercept**	316.182	18.200	<0.0001	0.515	0.124	0.079
lens.thick	4.487	3.116	0.157			
visual.systemv	15.380	10.180	0.139			

Table S6. The dependence of ocular media $\lambda_{T0.5}$ on cornea $\lambda_{T0.5}$ (N = 40 species).

**variable**	**estimate**	**SE**	**p.value**	**lambda**	**r2**	**r2.adj**
**intercept**	288.84	53.357	<0.0001	1	0.056	0.031
**cornea.trans**	0.227	0.156	0.148			

Table S7. The dependence of ocular media $\lambda_{T0.5}$ on lens $\lambda_{T0.5}$ (N = 40 species).

**variable**	**estimate**	**SE**	**p.value**	**lambda**	**r2**	**r2.adj**
**intercept**	159.844	24.176	<0.0001	0.288	0.727	0.72
**lens.trans**	0.585	0.069	**<0.0001**			

Table S8. The dependence of ocular media $\lambda_{T0.5}$ on cornea and lens $\lambda_{T0.5}$ and visual system (N = 40 species).

**variable**	**estimate**	**SE**	**p.value**	**lambda**	**r2**	**r2.adj**
**intercept**	-9.605	36.091	0.792	0.025	0.882	0.869
**cornea.trans**	0.569	0.113	**<0.0001**			
**lens.trans**	0.538	0.045	**<0.0001**			
**log10 (axial.length)**	-7.127	7.991	0.379			

Journal of Experimental Biology • Supplementary information
